# Supplementary material for: Effects of Ultra-High Pressure on Endogenous Enzyme Activities, Protein Properties, and Quality Characteristics of Shrimp (Litopenaeus vannamei) during Iced Storage
Source: Molecules. 2022 Sep 24;27(19):6302. doi: 10.3390/molecules27196302 (PMC9571125; doi:10.3390/molecules27196302)
Supplement: Supplementary file 1 [file molecules-27-06302-s001.zip › molecules-1911672-supplementary.pdf]

molecules

Supplementary Materials

Effects of Ultra-High Pressure on Endogenous Enzyme Activities, Protein Properties, and Quality Characteristics of Shrimp (*Litopenaeus Vannamei*) during Iced Storage

Chen Zhu †, Dexin Jiao †, Ying Sun, Lihang Chen, Siyu Meng [1,2], Xiaona Yu, Mingzhu Zheng, Meihong Liu, Jingsheng Liu and Huimin Liu*

Table S1. The parameters of ultra-high pressure.

Pressure （MPa）	Come-up Time (s)	Holding Time (s)	Decompression Time (s)	Min Pressure During Holding (MPa)	Max Pressure During Holding (MPa)
100	13	300	1	101	107
200	20	300	1	197	203
300	29	300	2	294	299
400	34	300	3	390	402
500	39	300	3	491	505

Table S2. The corresponding substrates, reaction buffers and stopping solution of cathepsin B, H and L.

Endogenous enzyme	Cathepsin B	Cathepsin H	Cathepsin L
corresponding substrates	Z-Arg-Arg-AMC	L-Arg-AMC	Z-Phe-Arg-AMC
reaction buffers	150 mM Bis-tris	150 mM Bis-tris	150 mM Bis-tris
	30 mM EDTA	30 mM EDTA	30 mM EDTA
	6 mmol/L DTT	6 mmol/L DTT	6 mmol/L DTT
	PH 6.0	PH 6.8	PH 5.5
stopping solution	1% SDS	1% SDS	1% SDS
	50 mM bis-Tris	50 mM bis-Tris	50 mM bis-Tris
	pH 7.0	pH 7.0	pH 7.0

Table S3. Effects of UHP treatment on textural properties of shrimp during iced storage.

	Pressure treatment	Storage time (days)					
		0	3	6	9	12	15
Hard-ness (N)	Control	244.29±20.28^{Ce}	439.32±3.98^{Da}	411.38±7.44^{Dab}	384.35±5.59^{Db}	334.26±4.3^{Dc}	275.85±6.71^{Dd}
	100 MPa	272.41±10.09^{Ce}	512.42±1.04^{Ca}	486.91±12.45^{Ca}	431.08±16.32^{Cb}	332.89±7.57^{Dc}	301.22±8.69^{Dd}
	200 MPa	301.02±21.17^{Be}	544.41±3.28^{Ba}	493.5±2.59^{Cb}	457.38±11.95^{Cc}	363.61±6.7^{Cd}	335.1±6.53^{Cd}
	300 MPa	314.08±12.74^{Be}	561.33±1.85^{Ba}	531.84±8.66^{Bb}	492.16±9.21^{Bc}	418.83±9.33^{Bd}	402.9±2.06^{Bd}
	400 MPa	317.98±9.36^{Be}	540.88±30.44^{BCa}	500.81±6.29^{Cb}	434.3±11.03^{Cc}	378.67±8.85^{Cd}	334.87±8.42^{Ce}
	500 MPa	482.56±17.29^{Af}	738.01±18.71^{Aa}	706.97±7.53^{Ab}	627.77±8.37^{Ac}	576.2±17.43^{Ad}	527.47±19.63^{Ae}
Sprin-gi-ness	Control	0.5±0.02^{ABa}	0.47±0.06^{Ba}	0.43±0.07^{Bb}	0.57±0.01^{Aa}	0.48±0.11^{Aa}	0.45±0.07^{Ba}
	100 MPa	0.47±0.04^{ABa}	0.53±0.09^{Ba}	0.51±0.06^{ABa}	0.56±0.02^{Aa}	0.46±0.02^{Aa}	0.45±0.08^{Ba}
	200 MPa	0.44±0.01^{Ba}	0.49±0.05^{Ba}	0.56±0.05^{Aa}	0.51±0.05^{Aa}	0.56±0.06^{Aa}	0.5±0.03^{ABa}
	300 MPa	0.59±0.01^{Aa}	0.53±0.03^{Ba}	0.55±0.03^{ABa}	0.56±0.06^{Aa}	0.57±0.05^{Aa}	0.48±0.01^{ABa}
	400 MPa	0.52±0.05^{ABa}	0.53±0.03^{Ba}	0.61±0.05^{Aa}	0.55±0.06^{Aa}	0.54±0.05^{Aa}	0.56±0.05^{ABa}
	500 MPa	0.57±0.04^{Abcd}	0.7±0.08^{Aa}	0.54±0.04^{ABd}	0.59±0.03^{Aabcd}	0.55±0.03^{Acd}	0.59±0.04^{Aabcd}
Gum-miness	Control	106.23±37.7^{Bc}	191.11±7.72^{Ba}	174.34±18^{Cab}	141.51±36.99^{Dabc}	137.34±14.59^{Babc}	119.02±12.31^{Dbc}
	100 MPa	103.03±33^{Bd}	234.34±28.49^{Ba}	210.91±13.14^{BCab}	166.63±19.77^{CDbc}	155.6±24.65^{Bbcd}	133.88±5.16^{CDcd}
	200 MPa	109.01±26.7^{Be}	255.57±12.97^{Ba}	206.75±5.66^{BCab}	195.55±37.81^{BCDbc}	133.68±15.2^{Bde}	139.71±14.34^{CDcde}
	300 MPa	149.44±31.64^{Bc}	248.96±41.66^{Ba}	236.55±35.79^{Ba}	232.5±26.7^{Bab}	180.18±4.28^{Bbc}	199.72±12.5^{Babc}
	400 MPa	149.12±7.43^{Bd}	265.5±19.45^{Ba}	264.8±19.04^{Ba}	208.45±22.9^{BCbc}	186.29±9.88^{Bbcd}	166.53±18.42^{BCDcd}
	500 MPa	216.85±8.62^{Ad}	446.49±24.71^{Aa}	409.58±18.73^{Aa}	341.91±25.5^{Ab}	270.16±43.44^{Acd}	270.81±17.7^{Acd}
Chewi-ness	Control	46.99±8.95^{Bc}	90.25±9.4^{Ca}	74.89±9.1^{Dabc}	75.43±8.02^{Cabc}	66.96±20.78^{Cab}c	53.61±3.9^{Dbc}
	100 MPa	49.24±16.85^{Bd}	114.41±9.69^{BCa}	107.6±10.65^{CDa}	92.95±14.87^{BCab}	71.03±13.4^{BCbcd}	59.89±13.4^{CDcd}
	200 MPa	48.24±12.81^{Bd}	123.58±6.29^{Ba}	114.74±10.76^{Ca}	100.87±26.86^{BCabc}	74.4±1.68^{BCbcd}	69.89±7.74^{BCDcd}
	300 MPa	76.87±4.51^{Bc}	131.87±18.66^{Ba}	129.44±18.73^{Ca}	124.04±18.01^{Bab}	101.87±10.73^{Babc}	95.38±5.17^{Bbc}
	400 MPa	77±6.52^{Be}	141.02±11.52^{Bab}	162.39±15.44^{Ba}	113.59±1.28^{Bbcd}	99.92±9.16^{Bcde}	93.54±9.32^{Bde}
	500 MPa	122.45±5.59^{Ae}	237.13±18.78^{Aa}	221.84±11.52^{Aab}	200.04±11.98^{Ab}	149.68±31.52^{Ade}	160.2±19.95^{Acd}
Shear force (N)	Control	10.45±1.18^{Ea}	10.45±1.18^{Ea}	11.25±1.13^{Da}	10.45±1.18^{Ea}	9.79±0.84^{Dc}	9.57±1.34^{Ea}
	100 MPa	13.16±1.22^{Da}	13.16±1.22^{Da}	12.95±0.22^{CDa}	13.16±1.22^{Da}	11.19±0.38^{Dc}	13.16±1.22^{Da}
	200 MPa	14.51±0.19^{CDa}	14.51±0.19^{CDa}	13.45±0.18^{Ca}	14.51±0.19^{CDa}	13.41±0.53^{Cd}	14.48±0.15^{CDa}
	300 MPa	15.84±0.83^{BCa}	15.51±0.47^{BCa}	14.59±0.41^{BCa}	15.51±0.47B^{Ca}	14.91±0.23^{Bd}	15.2±0.61^{BCa}
	400 MPa	17.81±0.94^{Ba}	17.11±0.6^{Ba}	15.81±0.32^{Bb}	17.11±0.6^{Ba}	16.27±1.73^{Cd}	16.84±0.17^{Aba}
	500 MPa	20.34±1.09^{Abcd}	22.23±0.72^{Aab}	20.48±0.61^{Abc}	23.66±1.25^{Aa}	19.04±0.26^{Ad}	18.36±0.51^{Ad}
Adhes-iveness	Control	-13.92±3.11^{ABab}	-11.72±1.07^{Aa}	-11.15±2.86^{ABa}	-19.66±2.11^{BCb}	-19.16±1.95^{Bb}	-13.81±3.21^{ABab}
	100 MPa	-12.76±2.9^{ABa}	-13.25±2.69^{ABa}	-9.82±2.44^{Aa}	-21.4±0.17^{Cb}	-12.04±3.84^{Aa}	-13.58±2.92^{ABa}
	200 MPa	-10.87±0.67^{Aa}	-8.98±0.97^{Aa}	-13.91±2.98^{ABa}	-14.44±3.08^{ABa}	-12.98±2.72^{ABa}	-15.44±3.94^{ABa}
	300 MPa	-14.85±3.09^{ABa}	-9.49±4.76^{Aa}	-11.94±2.36A^{Ba}	-9.82±4.38^{Aa}	-14.71±4.14^{ABa}	-13.14±0.77^{Aa}
	400 MPa	-18.09±4.18^{Bb}	-10.84±2.04^{Aa}	-16.82±0.95^{Bab}	-12.03±4.63^{Aab}	-15.39±1.62^{ABab}	-15.22±0.75^{ABab}
	500 MPa	-8.2±1.62^{Aa}	-19.15±3.03^{Bc}	-7.54±1.93^{Aa}	-10.36±3.32^{Aab}	-15.61±1.21^{ABbc}	-19.92±2.8^{Bc}
Cohes-iveness	Control	0.4±0.07^{ABa}	0.44±0.02^{Aa}	0.42±0.04^{Ba}	0.37±0.09^{Ca}	0.41±0.04^{ABa}	0.43±0.04^{Aa}
	100 MPa	0.48±0.05^{Aa}	0.49±0.01^{Aa}	0.43±0.02^{Ba}	0.39±0.03^{Ca}	0.47±0.08^{ABa}	0.45±0.03^{Aa}
	200 MPa	0.36±0.07^{Ba}	0.47±0.02^{Aa}	0.42±0.01^{Ba}	0.43±0.08^{BCa}	0.37±0.05^{Ba}	0.42±0.05^{Aa}
	300 MPa	0.45±0.06^{ABa}	0.44±0.08^{Aa}	0.44±0.06^{Ba}	0.54±0.06^{ABa}	0.43±0.02^{ABa}	0.5±0.03^{Aa}
	400 MPa	0.47±0.01^{ABa}	0.49±0.02^{Aa}	0.53±0.03^{ABa}	0.48±0.06^{ABCa}	0.49±0.04^{Aa}	0.5±0.04^{Aa}
	500 MPa	0.45±0.03^{ABb}	0.51±0.04^{Aab}	0.58±0.02^{Aa}	0.54±0.04^{Aab}	0.47±0.08^{ABab}	0.51±0.01^{Aab}
Resili-ence	Control	0.18±0.02^{Cb}	0.25±0.02^{Ba}	0.25±0.03^{Ba}	0.24±0.02^{BCab}	0.22±0.01^{Bab}	0.23±0.03^{Bab}
	100 MPa	0.21±0.02^{BCb}	0.26±0.03^{Bab}	0.27±0.03^{Ba}	0.22±0.01^{Cab}	0.25±0.04^{ABab}	0.24±0.01^{ABab}
	200 MPa	0.23±0.02^{BCb}	0.3±0.01^{ABa}	0.26±0.01^{Bab}	0.28±0.04^{ABCab}	0.24±0.01^{ABab}	0.24±0.03^{ABab}

300 MPa	0.21±0.01^{BCb}	0.29±0.05^{ABa}	0.28±0.04^{ABa}	0.3±0.01^{Aa}	0.26±0.01^{ABab}	0.28±0.02^{ABa}
400 MPa	0.25±0.02^{ABb}	0.3±0.01^{ABa}	0.29±0.02^{ABab}	0.29±0.02^{ABab}	0.27±0.02^{ABab}	0.25±0.05^{ABab}
500 MPa	0.29±0.02^{Aab}	0.34±0.04^{Ab}	0.33±0.02^{Aab}	0.32±0.03^{Aab}	0.28±0.01^{Ab}	0.29±0.01^{Aab}

Values are expressed as mean ± standard deviation (SD) (n = 3). Different capital letters in the same column indicate significantly different ($p < 0.05$). Different small letters in the same row indicate significantly different ($p < 0.05$).

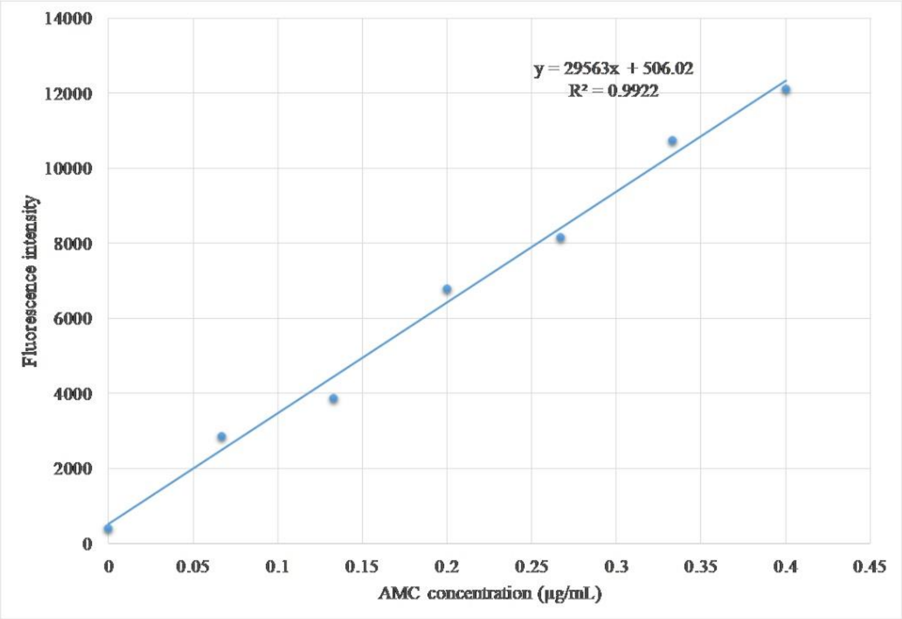

Figure S1. The standard curve of AMC.
